# Netrin-1 Promotes Synaptic Formation and Axonal Regeneration via JNK1/c-Jun Pathway after the Middle Cerebral Artery Occlusion

**DOI:** 10.3389/fncel.2018.00013

**Published:** 2018-02-13

**Authors:** Mouwei Zheng, Ronghua Chen, Hongbin Chen, Yixian Zhang, Jianhao Chen, Peiqiang Lin, Quan Lan, Qilin Yuan, Yongxing Lai, Xinhong Jiang, Xiaodong Pan, Nan Liu

**Affiliations:** ^1^Department of Neurology, Fujian Medical University Union Hospital, Fuzhou, China; ^2^Institute of Cerebral Vascular Disease of Fujian Province, Fuzhou, China; ^3^Key Laboratory of Brain Aging and Neurodegenerative Diseases, Fujian Key Laboratory of Molecular Neurology, Fujian Medical University, Fuzhou, China; ^4^Department of Rehabilitation, Fujian Medical University Union Hospital, Fuzhou, China

**Keywords:** Netrin-1, MCAO, JNK1, synaptic formation, axonal regeneration

## Abstract

As a secreted axon guidance molecule, Netrin-1 has been documented to be a neuroprotective factor, which can reduce infarct volume, promote angiogenesis and anti-apoptosis after stroke in rodents. However, its role in axonal regeneration and synaptic formation after cerebral ischemic injury, and the related underlying mechanisms remain blurred. In this study, we used Adeno-associated vectors carrying Netrin-1 gene (AAV-NT-1) to up-regulate the expression level of Netrin-1 in rats’ brain after middle cerebral artery occlusion (MCAO). We found that the up-regulated level of Netrin-1 and its receptor DCC promoted axonal regeneration and synaptic formation; the overexpression of Netrin-1 activated the JNK1 signaling pathway; these effects were partially reduced when JNK1 signaling pathway was inhibited by SP600125 (JNK specific inhibitor). Taken together, these findings suggest that Netrin-1 can facilitate the synaptic formation and axonal regeneration via the JNK1 signaling pathway after cerebral ischemia, thus promoting the recovery of neural functions.

## Introduction

Ischemic stroke, followed by long-term neurological impairment, is a leading cause of disability and death worldwide. At the early stage of cerebral ischemia, axon impairment often accompanies synaptic failure due to the structural damage of presynaptic and postsynaptic components by hypoxic injury (Hofmeijer and van Putten, 2012), thus resulting in permanent neurotransmission defect. Therefore, it is crucial to reduce the damage and promote axonal regeneration and synaptic formation in the injured area (penumbra) in the hope of recovering neural functions with efficient and effective treatment.

In the central nervous system (CNS) development and nerve injury, Netrin-1, a secreted protein, plays a role of guidance when in combination with specific receptors, especially Deleted in Colorectal Cancer (DCC). Therefore, Netrin-1 can serve as a candidate to ameliorate ischemia-induced neural damage. Studies have documented its favorable effects on axonal growth. When binding to the downstream transmembrane protein DCC, Netrin-1 is involved in the reorganization of actin cytoskeleton; it can promote axonal distribution and presynaptic differentiation of the optic tectum in Xenopus laevis; it can increase the axonal branching in mammalian fetal cortex (Dent et al., 2004) and is engaged in the development of human cerebral cortex (Harter et al., 2010); it has been demonstrated to enhance synaptic regeneration of cortical neurons by initiating synaptic assembly at the peak of postnatal synapse formation (Goldman et al., 2013); its tyrosine phosphorylation mediates Netrin-1 signaling in growth cone guidance by selectively interacting with the Src family kinases (Meriane et al., 2004; Ren et al., 2008); our previous study has found that rehabilitation training can up-regulate the expression of Netrin-1 and DCC in the peri-ischemic area in a rat MCAO model and improve the neural axonal growth and remodeling, thus promoting the functional recovery after the cerebral ischemia (Liu et al., 2011). However, little literature is available to shed light on Netrin-1’s mechanism in neural remodeling after the cerebral ischemia.

The JNK family is associated with a variety of physiological and pathological functions in the brain development, such as neuronal survival, migration, and regeneration. The suppression of the JNK signaling pathway by antisense RNA and JNK inhibitors can impair neurite outgrowth and axonal guidance. At the acute stage of stroke, the activation of this signaling pathway can cause inflammatory reaction and apoptosis in a rat MCAO model, but it remains obscure whether this pathway is involved in the neural remodeling process after the acute stage of stroke. JNK1 is one of the most important members of the JNK family, whose activation is related with its downstream proteins such as ATF-3, ATF-2, JunD and Elk-1. In a study of diffuse axonal injury, JNK1 activates the downstream factor c-Jun and in turn activates ATF-3, promoting axonal regeneration (Greer et al., 2011). The AP-1 transcription factor c-Jun is essential for axonal regeneration and neuronal cell death (Raivich et al., 2004). It is expressed during neurogenesis and its expression in the adult brain changes dramatically in response to the neuronal injury (Herdegen and Leah, 1998). Studies have reported that c-Jun mediates the axonal growth by affecting the expression of regeneration-induced molecules (Werner et al., 2000; Wynick et al., 2001; Jones et al., 2010; Lin and Chan, 2015) and that the phosphorylation of c-Jun is partly mediated by the JNKs (Davis, 2000). Another study documents that the degree of JNK1 activation can be improved when Netrin-1 binds to its receptor DCC and inhibited when JNK inhibitor SP600125 or an anti DCC antibody is employed (Qu et al., 2013). Therefore, we speculate that Netrin-1 can promote neural functional recovery after stroke by activating the JNK1 signaling pathway.

In this study, we established a rat middle cerebral artery occlusion (MCAO) model to investigate the role of Netrin-1 in the recovery of neural functions and the underlying regulatory mechanism. We found that the over-expression of AAV-mediated Netrin-1 facilitated synaptic formation and axonal regeneration after the MCAO via the JNK1 signaling pathway. This finding sheds new light on our knowledge of Netrin-1 and provides empirical evidence for targeting it in the clinical treatment of cerebral ischemia in the post-acute stage.

## Materials and Methods

### Adeno-Associated Virus Vector Production

Adeno-associated virus vectors used in this study were provided by Shanghai GeneChem Company (Shanghai, China). In brief, pAAV-NT-1 (with rat’s full-length Nt1 gene: NM_053731), pAAV-RC and pHelper plasmid were co-transfected into AAV-293 cells. The supernatant of AAV-293 cells was concentrated and purified after splitting, ultrafiltrating, and centrifugating. Virus titer was measured by Real-time Quantitative PCR (2.95E++12 v.g./ml). Empty vector (AAV-empty) was used as control. AAV expressing the gene for green fluorescent protein (AAV-GFP) was used to verify the success of transfection of vectors to the target region of the brain.

### Animals

A total of 96 adult male Sprague–Dawley rats (aged 2 months old and weighed 250–280 g) were used in this study. The animals were randomly divided into six groups: Sham group, AAV-empty group (receiving MCAO surgery and AAV-empty vector), AAV-NT-1 group (receiving MCAO surgery and AAV-NT-1 vector), AAV-NT-1+SP600125 group (receiving MCAO surgery and AAV-NT-1 vector and SP600125), AAV-empty+DMSO group (undergoing MCAO surgery and given AAV-empty vector and DMSO), AAV-empty+SP600125 group (undergoing MCAO surgery and given AAV-empty vector and SP600125). Results of the experiments performed on rats of AAV-empty+DMSO group and AAV-empty group were shown in the Supplementary Materials. Animals were raised in accordance with the principles prescribed in *The National Institute of Health Guide for the Care and Use of Laboratory Animals* (NIH Publications No. 80-23, revised in 1996). All study procedures were approved by the Institutional Animal Care and Use Committee of Fujian Medical University.

### MCAO Procedures

A permanent focal cerebral ischemia model was established by modifying Longa’s method (Longa et al., 1989). In brief, rats in the AAV-empty group, AAV-NT-1 group, AAV-NT-1+SP600125, AAV-empty+DMSO group and AAV-empty+SP600125 group underwent preoperative fasting of food (12 h) and water (4 h). The rats were then anesthetized with 10% chloral hydrate intraperitoneal at a dose of 0.3 ml/100 g. After anesthesia, the animals were fixed on the operating table and covered with a small heating blanket to keep the anus temperature at 37°C. After cleaning and disinfecting the operation area, a small incision was made in the midline of the neck. The right common carotid artery (CCA) (the proximal part) and external carotid artery (ECA) were isolated and ligated. Then, a microvascular clip was put in the CCA. A small cut was made in CCA (in the distal end of clip) and a 3.0 monofilament nylon suture was inserted along the CCA into the internal carotid artery, and after the clip removal, advanced until a mild resistance was felt (about 18 mm from the junction of ECA and ICA), thereby blocking the blood supply of the middle cerebral artery. Monofilament nylon wires were not inserted into the internal carotid artery in the Sham group. After the rats recovered from anesthesia, they were returned to their cages and allowed free access to food and water.

### Adeno-Associated Virus Transfer into the Rat Brain

A total of 1 × 10^10^ genome copies of AAV-Netrin-1, AAV-empty or AAV-GFP were injected into the rat brain in the peri-ischemic cortex and the ipsilateral striatum posterior to it 1h after the MCAO procedure. Briefly, Bregma was chosen as the origin of coordinate axis, and a tiny hole was drilled at the stereotaxic coordinates: (AP -0.2 mm, LM 2.5 mm). AAV-NT-1 or AAV-empty was injected into the brain tissue at a depth of 2.5 mm (cortex) and 4.5 mm (striatum) using a 10 μl microinjector at the rate of 0.2 μl/min. The needle remained in position for 15 min before removal to ensure a complete dispersion of the virus. To verify the success of transfection of these vectors, rats (*n* = 3) receiving AAV-GFP vector were sacrificed 3 days after the injection, brain sections (30 μm) were made with a Leica CM1850 cryostat (Leica Microsystems GmbH, Wetzlar, Germany) and a fluorescence microscope (Nikaon, DS-Ri2, Japan) was used to observe the GFP-positive cell in the peri-ischemic region.

In the AAV-NT-1+SP600125 group, the JNK inhibitor SP600125 (30 μg; Selleck Chemicals, United States), kept in amicrobic PBS containing 10 μl of 0.1% DMSO (Sigma, United States), was injected into the lateral ventricle 72 h after the MCAO. After the first injection, the same dose of drug was injected every 3 days. Rats in the AAV-empty group and AAV-NT-1 group received the same volume of vehicle (amicrobic PBS containing 10 μl of 0.1% DMSO) at each time point.

### Behavioral Testing

All rats underwent behavioral testing prior to MCAO procedure and at Day 1, 3, 7, 10, 14 after the MCAO (*n* = 18 in each group) by using modified Neurological Severity Scores (mNSS). The mNSS score is a multiple test of various aspects including motor (muscle status and abnormal movement), sensory (visual, tactile, and proprioceptive), reflex (pinna, corneal, and startle), and balance tests (Chen et al., 2001), aiming at a comprehensive evaluation of the severity of neurological deficit. The degree of defect was graded on a scale of 0 to 18 (normal score: 0; maximal deficit score: 18). Rats that scored 0 prior to the MCAO were qualified for subsequent studies.

### Western Blot

**FIGURE 1:**
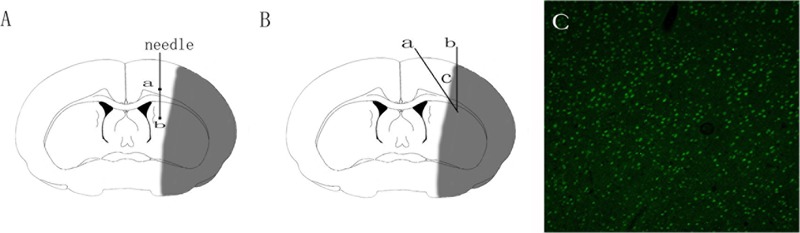
Schematic diagram of adeno-associated virus injection and brain tissue sampling. **(A)** Schematic diagram of adeno-associated virus injection. Square a represents cortex injection location, Square b striatum injection location. **(B)** Schematic diagram of the ischemic brain. The gray area in the diagram represents the ischemic core; area c encircled by Line a and b represents the peri-ischemic region that was dissected for western-bolt, TEM and immunofluorescence analysis. **(C)** GFP-positive cells in the cortex 3 days after the injection of adeno-associated virus.

The expressions of Netrin-1, DCC, p-DCC, JNK1, p-JNK1, c-Jun, p-c-Jun, PSD-95, SYN, NF-200, GAP-43, and MAP-2 in the peri-ischemic cortex in each group were analyzed by western blot. Rats were sacrificed at Day 14 after the MCAO (*n* = 3 each group). The tissues corresponding to the peri-ischemic cortex (**Figure 1A**) were dissected and then homogenized in the RIPA lysis buffer containing 1 mM PMSF (Beyotime, China). After grinding and incubation on ice for 30 min, the homogenate was centrifuged at 14000 ×*g* at 4°C for 15 min, and the supernatant was collected for protein analysis. Protein concentration was measured with BCA kit (Beyotime, China). Sodium dodecyl sulfate-polyacrylamide gel electrophoresis was performed by using the Bio-Rad Mini-Protean Tetra System (Bio-Rad, Hercules, CA, United States). An equal amount of total protein (30 μg) from each sample of the groups was separated by 10 or 12% SDS-PAGE and then subsequently transferred to PVDF membranes (Millipore, United States). Afterward, the membranes were blocked with a blocking solution (Beyotime, China) for 2 h and then incubated with the following primary antibodies at 4°C overnight: anti-Netrin-1 antibody, anti-DCC antibody, anti-p-DCC antibody, anti-JNK1 antibody, anti-p-JNK1 antibody, anti-c-Jun antibody, anti-p-c-Jun antibody (respectively, 1:800, 1:600, 1:600, 1:800, 1:800, 1:600, 1:600, Santa Cruz, United States), anti-PSD-95, anti-SYN, anti-GAP-43, anti-MAP-2 (respectively, 1:1000, 1:1000, 1:800, 1:1000, Abcam, United Kingdom), anti-NF-200, anti-GAPDH (respectively, 1:500, 1:1000, Boster Biological Technology, China). After washing with PBST (pH 7.4) for 15 min, the membranes were then incubated with IgG-HRP secondary antibodies (1:8000, Abcam, United Kingdom) at room temperature for 2 h. The signal was then detected with ECL reagent kits (Beyotime, China) and intensity of each band was analyzed with the Image J software (1.46r). The relative expression levels of proteins were, respectively, normalized to the internal control.

### Immunofluorescence Staining

At Day 14 after the MCAO, rats (*n* = 4 in each group) were anesthetized and perfused transcardially with phosphate-buffered saline (PBS, at 4°C, pH 7.4) and subsequently with PBS containing 4% paraformaldehyde (pH 7.4) at 4°C. The brains were removed and post-fixed by immersion in PBS containing 4% paraformaldehyde (pH 7.4) at 4°C for 6 h. After post-fixation, these brains were placed in PBS containing 20% sucrose (pH 7.4) at 4°C until they sank and then placed in PBS containing 30% sucrose (pH 7.4) at 4°C until they sank again. Coronal sections in 8-μm thickness were cut with a Leica CM1850 cryostat (Leica Microsystems GmbH, Wetzlar, Germany) and collected on glass slides. Immunofluorescence staining was used to assess the expression of NF-200, GAP-43, and MAP-2. Double immunofluorescence staining was used to measure the co-localization of p-JNK1 and DCC. Frozen sections were rewarmed at room temperature for 60 min and then washed with PBS (PH 7.4). The sections were then incubated with 0.25% TritonX-100 (Amresco, Solon, United States) for 30 min. Subsequently, the sections were washed again and blocked with 5% Donkey Serum at 37°C for 2 h. Then the slices were incubated with the following primary antibodies at 4°C overnight: anti-NF-200 antibody (1:100, Boster Biological Technology, China), anti-GAP-43 antibody, and anti-MAP-2 antibody (respectively, 1:1000 and 1:800, Abcam, United States). To co-localize the expression of the DCC and p-JNK1, the following primary antibodies were used: anti-DCC (1:500, Santa Cruz, United States), anti p-JNK1 (1:200, Santa Cruz, United States) and DAPI (5 μg/ml, Beyotime, United States). After washing in PBS (pH 7.4) for 30 min, secondary anti-bodies were then applied at room temperature for 2 h: Cy3IgG (1:400, Jackson Immunoresearch, United States) and Dylight488 IgG (1:400, Jackson Immunoresearch, United States). After washing in PBS (pH 7.4) for 15 min, sections were incubated with DAPI (5 μg/ml, Beyotime, United States) at room temperature for 20 min. Negative controls were routinely performed with each staining experiment by omitting the primary antibodies. Sections were then mounted and examined under a Zeiss LSM 510 confocal microscope (Carl Zeiss, Jena, Germany), and attention was mainly focused on the expression patterns of the interested proteins in the peri-ischemic cortex (as **Figure 1B** shows). For each group and experiment, 3∼5 target fields were randomly selected and the average fluorescence intensity was analyzed with the Image J software. All trials were repeated three times.

### Transmission Electron Microscope Detection

At Day 14 after the MCAO, after anesthesia, rats (four animals per group) were transcardially perfused with PBS (pH 7.4), and subsequently with PBS (pH 7.4) containing 1.5% glutaraldehyde and 3% paraformaldehyde (pH 7.4). The brain was rapidly removed, and 1 mm^3^ tissue was dissected from the peri-ischemic cortex. The tissues were fixed in 1% osmium acid and 1.5% ferrocyanide for 1.5 h, and dehydrated with a gradient series of alcohol and acetone. The samples were then embedded with Epon618 epoxy resin. The tissue blocks were then cut into semi-thin sections (80 nm), respectively, stained with uranyl acetate and lead citrate for 5 min and examined under a lightmicroscope. Selected areas from semi-thin sections were then cut into thin sections. After uranyl acetate/lead citrate double staining, neurons and ultra-structures were observed under a Philips EM208 transmission electron microscope. Synapses are classified as excitatory synapses and inhibitory synapses. Excitatory synapses with prominent post-synaptic densities and relatively wide synaptic clefts (approx. 18∼20 nm), while inhibitory synapses with pre- and post-synaptic densities of equal thickness and narrower synaptic clefts (approximately 12 nm) (High et al., 2015). In this study, excitatory synapses were examined for synaptic modification. Approximately 70 synapses of each group were examined, and four animals were included in each group. The width of synaptic cleft (the mean of three values including the largest, middle, and smallest), the total area of PSD, the thickness of PSD at the thickest part, the length of the active zones, and the curvature of the synaptic interface were examined using image pro plus 6.0 (Media Cybernetics, United States).

### Data Analysis

All data were analyzed using IPP 6.0 (Media Cybernetics, United States) and SPSS 19.0 (IBM, United States) statistical software. Data were presented as means ± SEM. Statistical analysis for multiple comparisons was performed by one-way ANOVA, followed by Turkey’s *post hoc* tests. For the comparison between two groups, data were analyzed with standard two-tailed unpaired *t*-tests. *P*-value less than 0.05 was considered statistically significant.

## Results

### Adeno-Associated Virus Mediates Netrin-1 Over-Expression in the Peri-Ischemic Area after the MCAO

The transfection of AAV-GFP vehicle at Day 3 after the injection was observed under the fluorescence microscope by detecting the GFP-positive cells. The observation showed that the AAV vehicle was successfully transferred into nerve cells in the peri-ischemic area (**Figure 1C**). To ensure that the over-expression of the Adeno-associated-virus-mediated Netrin-1 matched the over-expression standard of experimental proteins, we analyzed the expression of Netrin-1 by Western-blot at Day 14 after the injection (**Figure 3A**). The analysis demonstrated that the expression of Netrin-1 in the AAV-NT-1 group and AAV-NT-1+SP600125 group was much higher than that of the AAV-empty group (respectively, 1.0400 ± 0.04726 vs. 0.3400 ± 0.03512, *p* < 0.001; 0.9967 ± 0.04096 vs. 0.3400 ± 0.03512, *p* < 0.001) (**Figure 3C**).

### Netirn-1 Activates the JNK1/c-Jun Signaling Pathway after the Stroke

**FIGURE 2:**
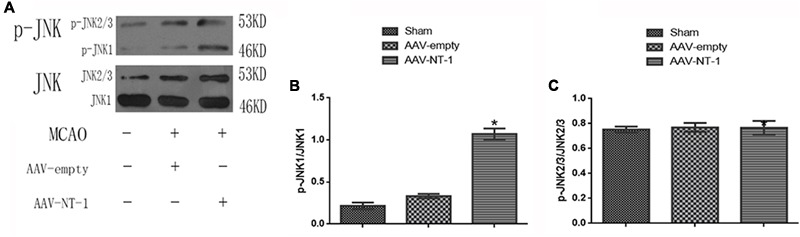
Effects of Netrin-1 overexpression on the JNK signaling pathway after the MCAO. **(A)** The image of western-blot analysis for p-JNK/JNK. Rats in each group were sacrificed at Day 14 after the MCAO. **(B)** Western-blot analysis of p-JNK1 (*n* = 3), ^∗^*p* < 0.05, as compared with the AAV-empty group. **(C)** Western-blot analysis of p-JNK2/3, No significant difference was found among the groups.

**FIGURE 3:**
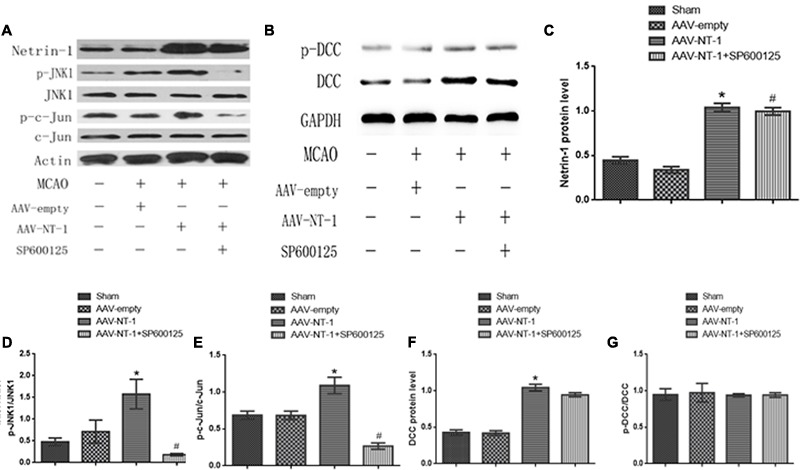
Effects of Netrin-1 overexpression on the DCC/JNK1/c-Jun signaling pathway after the MCAO. **(A)** The representative image of western-blot analysis for Netrin-1, p-JNK1/JNK, p-c-Jun/c-Jun. Rats in each group were sacrificed at Day 14 after the MCAO. **(B)** The image of western-blot analysis for p-DCC/DCC. **(C)** Western-blot analysis of Netrin-1 (*n* = 3), ^∗^*p* < 0.001, as compared with the AAV-empty group, ^#^*p* < 0.001, as compared with the AAV-empty group. **(D)** Western-blot analysis of p-JNK1 (n = 3), ^∗^*p* < 0.05, as compared with the AAV-empty group, ^#^*p* < 0.01, as compared with the AAV-NT-1 group. **(E)** Western-blot analysis of p-C-Jun (*n* = 3), ^∗^*p* < 0.01, as compared with the AAV-empty group, ^#^*p* < 0.001, as compared with the AAV-NT-1 group. **(F)** Western-blot analysis of DCC (*n* = 3), ^∗^*p* < 0.001, as compared with the AAV-empty group. **(G)** Western-blot analysis of p-DCC (*n* = 3).

To elucidate whether Netrin-1 activated the JNK signaling pathway after the MCAO, we first detected the activity of the JNK signaling pathway in the Sham group, AAV-empty group, and AAV-NT-1 group. We found that compared with the AAV-empty group, Netrin-1 overexpression only activated the JNK1 signaling pathway (0.3333 ± 0.02603 vs. 1.0767 ± 0.06741, *p* < 0.001) (**Figure 2B**), but not the JNK2/3 signaling pathway (**Figure 2C**). To further explore the change of JNK1 signaling pathway, we analyzed the level of p-JNK1 and p-c-Jun in each group by Western blot (**Figure 3A**). The analysis showed that the expression of p-JNK1 and p-c-Jun in the peri-ischemic area in the AAV-NT-1 group was, respectively, higher than that in the AAV-empty group (p-JNK1, 0.7133 ± 0.26535 vs. 1.5767 ± 0.33795, *p* < 0.05; c-Jun, 0.6833 ± 0.05696 vs. 1.0900 ± 0.11015, *p* < 0.01) (**Figures 3D,E**). Moreover, in rats treated with both AAV-NT-1 and SP600125, the expression of p-JNK1 and p-c-Jun was obviously decreased when compared with rats treated with AAV-NT-1 only (p-JNK1, 0.1,33 ± 0.02603 vs. 1.5767 ± 0.33795, ^#^*p* < 0.01; p-c-Jun, 0.2667 ± 0.04256 vs. 1.0900 ± 0.11015, ^#^*p* < 0.001) (**Figures 3D,E**). These findings suggest that Netrin-1 activates the JNK1/c-Jun signaling pathway after the MCAO while the treatment of SP600125 effectively reduces the activation.

### Netrin-1 Receptor DCC Is Up-regulated by Netrin-1 Overexpression and Co-localizes with p-JNK1

**FIGURE 4:**
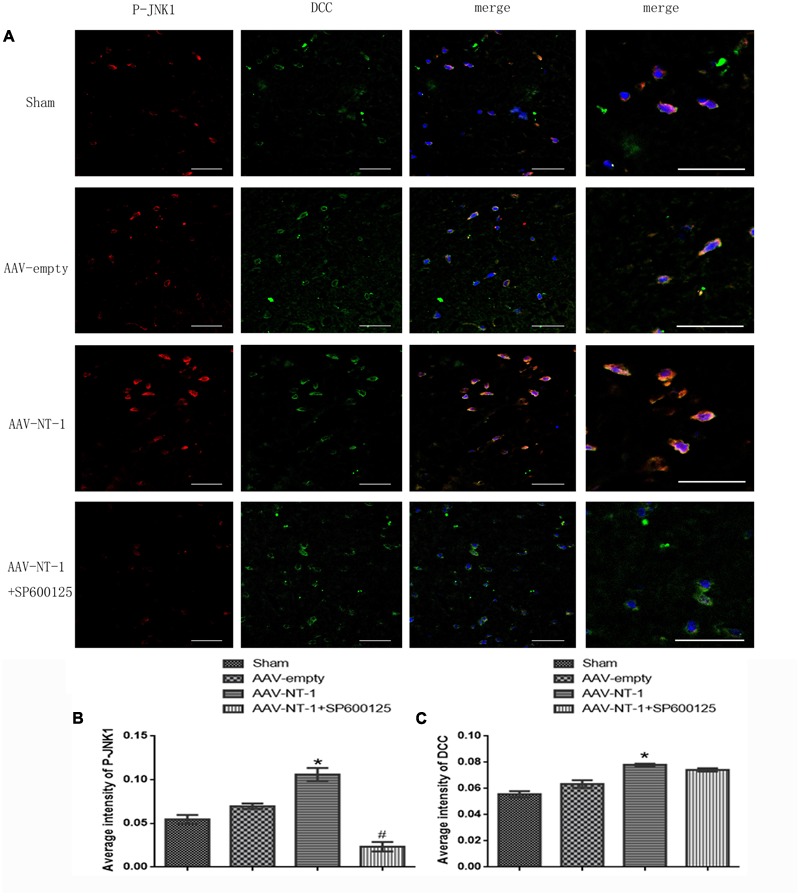
Co-localization of p-JNK1 and DCC. **(A)** Representative images of each group were shown. Rats in each group were sacrificed at Day 14 after the MCAO. Columns displays the expression of p-JNK1 (red), DCC (green), DAPI (blue); the right column displays the co-localization of p-JNK1, DCC and DAPI. White arrow points at distinct areas of co-localization. Scale bars = 50 μm. **(B)** Immunofluorescence analysis of p-JNK1, ^∗^*p* < 0.01, as compared with the AAV-empty group; ^#^*p* < 0.05, as compared with the AAV-NT-1+SP600125 group. **(C)** Immunofluorescence analysis of DCC (*n* = 3), ^∗^*p* < 0.05, as compared with the AAV-empty group; ^#^*p* < 0.05, as compared with the AAV-NT-1 group.

As a strong attractive receptor of Netrin-1, DCC is essential in commissural axon guidance and outgrowth. To determine the role of DCC in Netrin-1-meditated neural reconstruction, we analyzed the level of DCC and p-DCC by western-blot (**Figure 3B**). The results showed that Netrin-1 overexpression in the AAV-NT-1 group strongly up-regulated the expression of DCC when compared with that of the AAV-empty group (1.0430 ± 0.04765 vs. 0.4193 ± 0.03330, *p* < 0.001) (**Figure 3F**). Additionally, DCC level in the AAV-NT-1+SP600125 group was also markedly higher than that of the AAV-empty group (0.9447 ± 0.02826 vs. 0.4193 ± 0.03330, *p* < 0.001) (**Figure 3F**) while no significant difference was found between the AAV-NT-1 group and AAV-NT-1+SP600125, which indicates that the inhibition of the JNK1 pathway may have little effect on the expression of DCC. However, we found that Netrin-1 overexpression did not change the relative level of p-DCC (**Figure 3G**) and treating rats with DMSO or SP600125 separately did not affect the expression of DCC and p-DCC (**Supplementary Figures S1B,C**). The result of immunofluorescence co-localization of DCC and p-JNK1 showed that DCC largely merged with p-JNK1 in the peri-ischemic area (**Figure 4A**). The expression level of p-JNK1 and DCC was, respectively, in line with the results of western-blot (p-JNK1, AAV-NT-1 group vs. AAV-empty group, 0.106000 ± 0.007420 vs. 0.069700 ± 0.003100, *p* < 0.01; AAV-NT-1+SP600125 group vs. AAV-NT-1 group, 0.023300 ± 0.005327 vs. 0.106000 ± 0.007420, *p* < 0.0001) (**Figure 4B**) (DCC, AAV-NT-1 group vs. AAV-empty group, 0.078000 ± 0.000970 vs. 0.063360 ± 0.002920, *p* < 0.01) (**Figure 4C**). Altogether, these findings suggest that the over-expression of Netrin-1 up-regulates the level of its axonal attractive receptor DCC and facilitates axon attraction and neurite outgrowth by activating the JNK1 signaling pathway.

### Netrin-1 Promotes the Recovery of Neural Functions via the JNK1 Signaling Pathway after the MCAO

**FIGURE 5:**
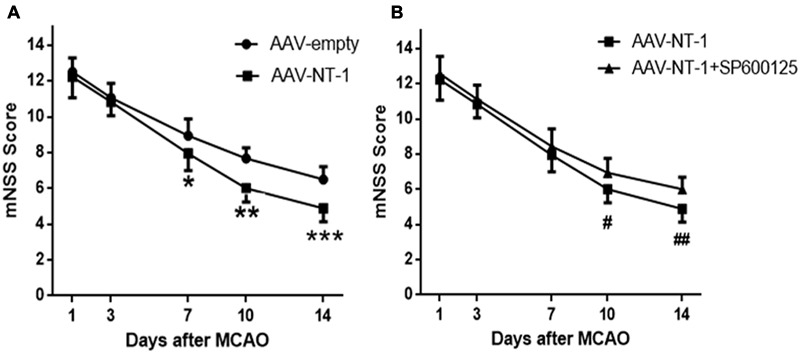
Behavioral testing (mNSS). **(A)** Quantitative analysis of mNss scores. Rats in the AAV-NT-1 group reported significantly lower scores at Day 7, 10, and 14, ^∗^*p* < 0.01, ^∗∗^*p* < 0.001, ^∗∗∗^*p* < 0.001, respectively, as compared with the AAV-empty group at the corresponding time points. **(B)** Quantitative analysis of mNss scores. Rats in the AAV-NT-1 group reported much lower scores at Day 10 and 14, ^#^*p* < 0.001, ^##^*p* < 0.001, respectively, as compared with the AAV-NT-1+SP600125 group at the corresponding time points.

To assess the effects of Netrin-1 and the role of JNK1 in neural functional recovery macroscopically, mNSS score was used to detect neurological deficits in motor, sensor, reflex, and equilibrium sense. The mNSS scores of the AAV-empty group, AAV-NT-1 group and AAV-NT-1+SP600125 group were summarized in **Figure 2**. No respective significant difference among the three groups was evident at Day 1 and Day 3. Compared with the AAV-empty group, rats in the AAV-NT-1 group received a lower score at Day 7 (7.94 ± 0.938 vs. 8.89 ± 0.900, ^∗^*p* < 0.01), Day 10 (6.00 ± 0.767 vs. 7.67 ± 0.594, ^∗∗^*p* < 0.001) and Day 14 (4.89 ± 0.758 vs. 6.50 ± 0.707, ^∗∗∗^*p* < 0.001) (**Figure 5A**). Of note, compared with the AAV-NT-1+SP600125 group, rats in the AAV-NT-1 group received a much lower score at Day 10 (6.00 ± 0.767 vs. 6.94 ± 0.802, ^#^*p* < 0.001) and Day 14 (4.89 ± 0.758 vs. 6.00 ± 0.686, ^##^*p* < 0.001) (**Figure 5B**), but no significant difference was found between them at Day 7. These findings suggest that Netrin-1 promotes neural functional recovery in the sub-acute stage partly via the activation of JNK1/c-Jun pathway and that suppression of this pathway may reduce these benefits.

### Netrin-1 Promotes Synaptic Formation via the JNK1/c-Jun Signaling Pathway after the MCAO

**FIGURE 6:**
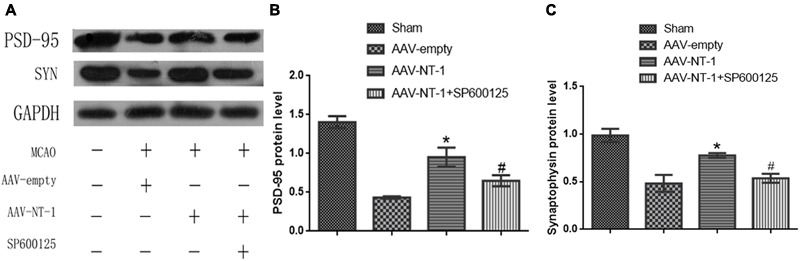
Effects of Netrin-1 overexpression and/or JNK inhibition on the synaptic formation after the MCAO. **(A)** The representative image of western-blot analysis for PSD-95 and synaptophysin. Rats in each group were sacrificed at Day 14 after the MCAO. **(B)** Western-blot analysis of PSD-95 (*n* = 3), ^∗^*p* < 0.01, as compared with the AAV-empty group; ^#^*p* < 0.05, as compared with the AAV-NT-1+SP600125 group. **(C)** Western-blot analysis of synaptophysin (*n* = 3), ^∗^*p* < 0.05, as compared with the AAV-empty group; ^#^*p* < 0.05, as compared with the AAV-NT-1 group.

To further explore the effect of Netrin-1 on synaptic formation and its intrinsic mechanism after the experimental stroke, we first analyzed the expression of pre-synaptic protein, SYN, and post-synaptic protein, PSD-95 (**Figure 6A**). Western blot analysis showed that the expression of PSD-95 and SYN both increased significantly in the AAV-NT-1 group when compared with that of the AAV-empty group (respectively, 0.9533 ± 0.12252 vs. 0.4333 ± 0.01453, ^∗^*p* < 0.01; 0.7767 ± 0.02333 vs. 0.4833 ± 0.08838, ^∗^*p* < 0.05) (**Figures 6B,C**). However, the expression of these two proteins both decreased in the MCAO+AAV-NT-1+SP600125 group when compared with that of the MCAO+AAV-NT-1 group (0.6500 ± 0.07024 vs. 0.9533 ± 0.12252, ^#^*p* < 0.05 for PSD-95; 0.5367 ± 0.04631 vs. 0.7767 ± 0.02333, ^#^*p* < 0.05 for SYN) (**Figures 6B,C**). Furthermore, results also showed that the injection of DMSO had no effects on the expression of PSD-95 and synaptophysin. The inhibition of JNK1 signaling pathway reduced the expression of PSD-95 and SYN (in a respective comparison with the AAV-empty group: 0.6600 ± 0.04933 vs. 0.8267 ± 0.02333, ^∗^*p* < 0.05; 0.6667 ± 0.04485 vs. 0.9600 ± 0.04041, ^∗^*p* < 0.01; in a respective comparison with the AAV-empty+DMSO group: 0.6600 ± 0.04933 vs. 0.8300 ± 0.01528, ^∗^*p* < 0.05; 0.6667 ± 0.04485 vs. 0.9700 ± 0.07211, ^∗^*p* < 0.01) (**Supplementary Figures S2B,C**). The results demonstrate that Netrin-1 promotes both pre-synaptic and post-synaptic formation partly via the activation of the JNK1/c-Jun signaling pathway after the cerebral ischemia.

### Netrin-1 Promotes Neural Ultrastructural Modification via the JNK1/c-Jun Signaling Pathway after the MCAO

**FIGURE 7:**
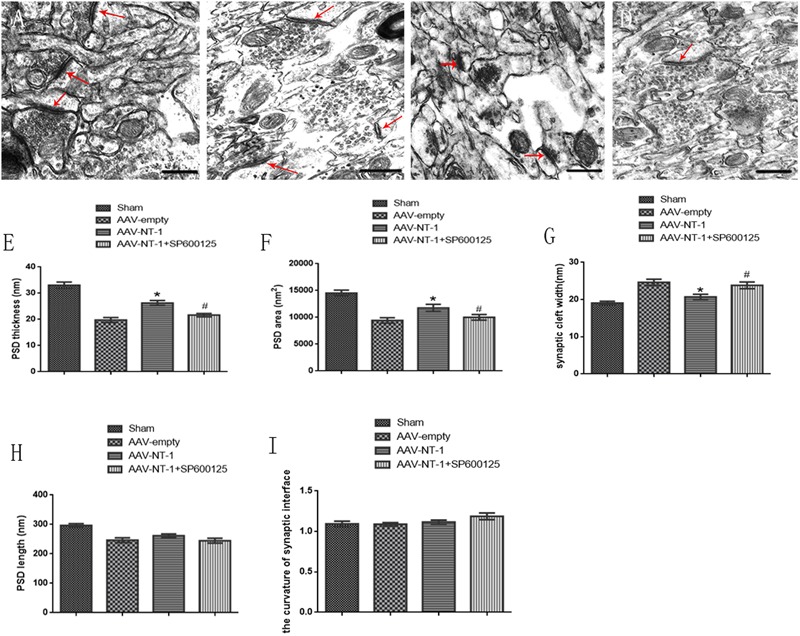
Effects of Netrin-1 overexpression and/or JNK inhibition on the neural ultra-structural modification after the MCAO. Representative images of each group were shown. Rats in each group were sacrificed at Day 14 after the MCAO. **(A)** Sham group. **(B)** AAV-empty group. **(C)** AAV-NT-1 group. **(D)** AAV-NT-1+SP600125 group. Red arrows point at the post-synaptic area. Scale bars = 400 nm. **(E)** Analysis of PSD thickness (*n* = 30), ^∗^*p* < 0.001, as compared with AAV-empty group, ^#^*p* < 0.01, as compared with the AAV-empty group. **(F)** analysis of PSD area (n = 30), ^∗^*p* < 0.01, as compared with AAV-empty group, ^#^*p* < 0.05, as compared with AAV-empty group. **(G)** analysis of synaptic cleft width (*n* = 30), ^∗^*p* < 0.001, as compared with AAV-empty group, ^#^*p* < 0.01, as compared with AAV-empty group. **(H)** analysis of PSD length (*n* = 30), no significant difference in PSD length among the groups. **(I)** analysis of PSD length (*n* = 30), no significant difference in the curvature of synaptic interface among the groups.

We used TEM to observe the morphological changes of the synapses in each group (**Figures 7A–D**). We found that hypoxic and ischemic injury caused ultrstructural damage to axons and synapses. Ultrastructures in the AAV-empty (**Figure 7B**) and AAV-NT-1+SP600125 (**Figure 7D**) group were fuzzy and disorganized while in the AAV-NT-1 group (**Figure 7C**), both axonal and synaptic ultrastructures were clear and relatively intact. These ultra-structural analysis of the synapses in the penumbra region revealed that when compared with those of the AAV-empty group, the AAV-NT-1 treatment increased the PSD thickness and PSD area (respectively, 26.9245 ± 0.92616 vs. 19.7727 ± 0.93745, ^∗^*p* < 0.001; 11757.83853 ± 673.123819 vs. 9432.20910 ± 487.107488, ^∗^*p* < 0.01) (**Figures 7E,F**), and decreased synaptic cleft width (20.7141 ± 0.68925 vs. 24.6432 ± 0.85151, ^∗^*p* < 0.001) (**Figure 7G**). Moreover, the treatment with SP600125 blocked the NT-1 over-expression-induced increase of PSD thickness (21.6397 ± 0.64356 vs. 26.9245 ± 0.92616, ^#^*p* < 0.01) (**Figure 7E**), PSD area (10015.70697 ± 524.088754 vs. 11757.83853 ± 673.123819, ^#^*p* < 0.05) (**Figure 7F**) and decreased the width of the synaptic cleft (23.8325 ± 0.90889 vs. 20.7141 ± 0.68925, ^#^*p* < 0.01) (**Figure 7G**). Interestingly, no significant difference in the PSD length (**Figure 7H**) and the curvature of the synaptic interface (**Figure 7I**) was found among the groups. Taken the results of Western-blot, immunofluorescence and neural ultrastructural analysis together, we speculate that Netirn-1 effectively promotes synaptic formation and axonal regeneration after the cerebral ischemia and the activation of JNK1/c-Jun pathway is the vital factor in these processes.

### Netrin-1 Facilitates Axonal Regeneration via the JNK1 Signaling Pathway after the MCAO

**FIGURE 8:**
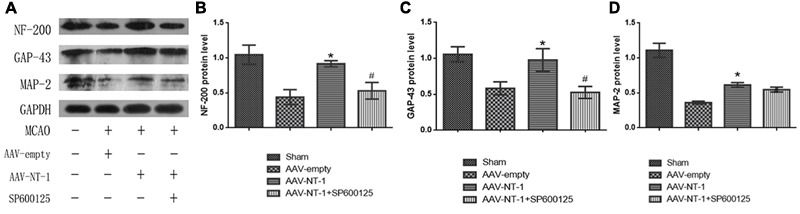
Effects of Netrin-1 overexpression and/or JNK inhibition on the axonal regeneration after the MCAO. **(A)** The representative images of western-blot analysis for NF-200, GAP-43 and MAP-2. Rats in each group were sacrificed at Day 14 after the MCAO. **(B)** Western-blot analysis of NF-200 (*n* = 3), ^∗^*p* < 0.01, as compared with the AAV-empty group, ^#^*p* < 0.05, as compared with the AAV-empty group. **(C)** Western-blot analysis of GAP-43 (*n* = 3), ^∗^*p* < 0.05, as compared with the AAV-empty group, ^#^*p* < 0.01, as compared with the AAV-NT-1 group. **(D)** Western-blot analysis of MAP-2 (*n* = 3), ^∗^*p* < 0.05, as compared with the AAV-empty group.

**FIGURE 9:**
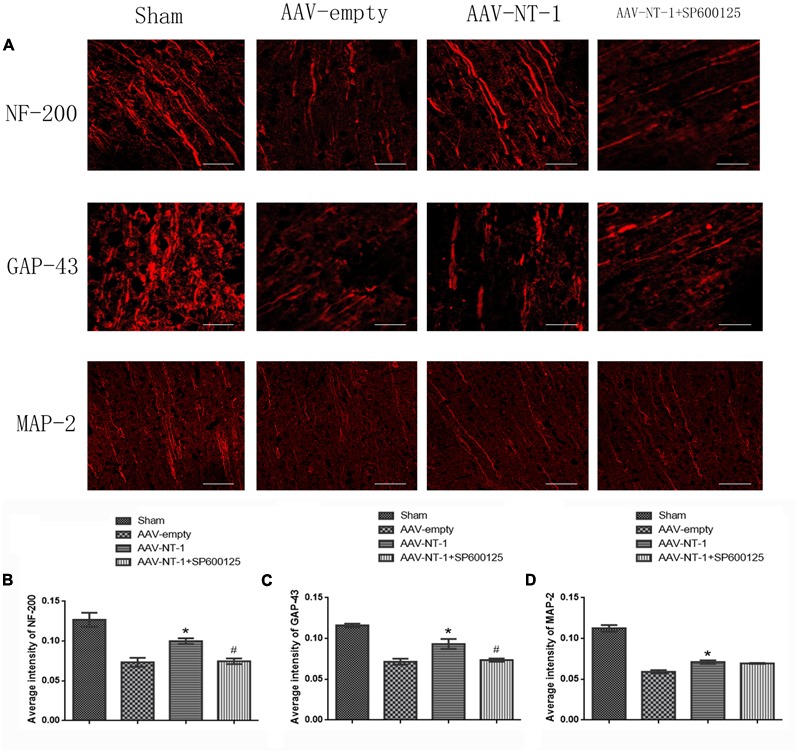
Images of immunofluorescence staining in each group. **(A)** Pictures represent the expression of NF-200, GAP-43 and MAP-2, respectively. Rats in each group were sacrificed at Day 14 after the MCAO. Scale bar = 50 μm. **(B)** Immunofluorescence analysis of NF-200 (*n* = 3), ^∗^*p* < 0.05, as compared with the AAV-empty group, ^#^*p* < 0.05, as compared with the AAV-NT-1 group. **(C)** Immunofluorescence analysis of GAP-43 (*n* = 3), ^∗^*p* < 0.01, as compared with the AAV-empty group, ^#^*p* < 0.01, as compared with the AAV-NT-1 group. **(D)** Immunofluorescence analysis of MAP-2 (*n* = 3), ^∗^*p* < 0.05, as compared with the AAV-empty group.

To determine the role of Netrin-1 in axonal regeneration after the MCAO, we analyzed the expression level of NF-200, MAP-2, and GAP-43 (**Figure 8A**). The analysis showed that when compared with those of the AAV-empty group, Netrin-1 up-regulated the expression of these axonal-structure-associated proteins (NF-200, 0.90167 ± 0.04333 vs. 0.4400 ± 0.14742, ^∗^*p* < 0.01; GAP-43, 0.90767 ± 0.15762 vs. 0.5833 ± 0.09025, ^∗^*p* < 0.05; MAP-2, 0.6133 ± 0.03844 vs. 0.3600 ± 0.02082, ^∗^*p* < 0.05) (**Figures 8B–D**). Additionally, these increased expressions were partly reversed by SP600125 (NF-200, 0.5300 ± 0.11930 vs. 0.9017 ± 0.04333, ^#^*p* < 0.05; GAP-43, 0.5267 ± 0.08413 vs. 0.90767 ± 0.15762, ^#^*p* < 0.01) (**Figures 8B,C**). Interestingly, no difference in MAP-2 expression was evident between the AAV-NT-1 group and AAV-NT-1+SP600125 group (0.6133 ± 0.03844 vs. 0.4000 ± 0.03125, ^#^*p* > 0.05) (**Figure 8D**). The results also showed that the injection of DMSO had no effects on the expression of NF-200, GAP-43 and MAP-2. The inhibition of the JNK1 signaling pathway reduced the expression of GAP-43 only (compared with the AAV-empty group, 0.6633 ± 0.02906 vs. 0.9033 ± 0.04910, ^∗^*p* < 0.01; compared with the AAV-empty+DMSO group, 0.6633 ± 0.02906 vs. 0.8500 ± 0.05033, ^∗^*p* < 0.05) (**Supplementary Figure S3B**). Furthermore, the results of immunofluorescence staining (**Figure 9A**) (the analysis of average intensity) were consistent with those of western blot analysis (compared with the AAV-empty group: NF-200, 0.100007 ± 0.003530 vs. 0.073330 ± 0.005610, ^∗^*p* < 0.05; GAP-43, 0.093300 ± 0.006130 vs. 0.071600 ± 0.003640, ^∗^*p* < 0.01; MAP-2, 0.071000 ± 0.002140 vs. 0.059200 ± 0.001940, ^∗^*p* < 0.05) (**Figures 9B–D**) (compared with the AAV-NT-1 group: NF-200, 0.074700 ± 0.003480 vs. 0.100007 ± 0.003530, ^#^*p* < 0.05; GAP-43, 0.073500 ± 0.001910 vs. 0.093300 ± 0.006130, ^#^*p* < 0.01) (**Figures 9B,C**).

## Discussion

The current study employed a rat MCAO model to investigate the role of Netrin-1 in the recovery of neural functions. It found that the AAV-induced over-expression of Netrin-1 increased the expression of Netrin-1 receptor DCC and activated the JNK1/c-Jun pathway in the ischemic penumbra. The study also found that Netrin-1 promoted neural functional recovery, synaptic formation and axonal regeneration in the subacute stage of stroke, and that the inhibition of JNK1/c-Jun pathway partly abolished these effects.

Axonal injury and synaptic failure after stroke are primary factors for the defection or even loss of neural transmission, which can induce permanent neurological deficits (Di Giovanni, 2009; Hofmeijer and van Putten, 2012). Neuroplastic processes occur spontaneously and last for months after stoke, trying to repair or even partially establish a new neural circuit. However, these complex processes are full of mysteries and are usually not efficient enough to remedy the nerve damage. Therefore, in stroke treatment, it is of great significance to clarify the half-known molecular mechanism and find new appropriate pathways to enhance these processes.

Netrin-1, known as highly conserved laminin-associated secret proteins (Serafini et al., 1994), is widely expressed in the CNS of vertebrates. It can either attract or repel axons depending on which receptor it binds to (Barallobre et al., 2005). In the development of embryonic nervous system, Netrin-1 acts as a key factor for axonal guidance, cell migration, morphogenesis and angiogensis. Netrin-1 binding with its receptor DCC can regulate synaptic plasticity and promote axonal outgrowth in both developmental and mature CNS (Horn et al., 2013). Previous studies demonstrate that Netrin-1 facilitates angiogenesis and long-term neurological recovery (Lu et al., 2011, 2012), decreases the infarct size (Ding et al., 2014; Yang et al., 2017), improves spatial memory and synaptic plasticity (Bayat et al., 2012), and promotes anti-apoptosis after stroke. Above all, Netrin-1 can facilitate neural functional recovery after hypoxic ischemic injury in versatile ways. However, the underlying molecular mechanisms of these neuroprotective effects are rarely elucidated. Our previous study found that treadmill exercise significantly increased the expression of Netrin-1 and its receptor DCC after the acute stage of cerebral ischemia injury. The expression level of these two proteins started to increase at Day 4, peaked at Day 14 and then decreased slowly. Immunofluorescence analysis showed that the interaction between Netrin-1 and DCC might play an important role in forming new neural circuits. So, we hypothesize that Netrin-1 binding to DCC may participate in the complicated process of neural regeneration after a cerebral ischemia.

AAV-gene vectors have been widely used in various nerve injury models for its efficient transduction ability, persistent, and steady expression of target proteins with low toxicity (Xiao et al., 1996). In this study, we chose AAV to mediate the expression of Netrin-1. We observed the empty-positive cells in the peri-ischemia region and detected the expression of Netrin-1 to ensure that AAV-NT-1 was transferred to the target area and functioned well.

JNKs, as a subfamily of MAPKs, are highly expressed in the CNS and involved in various physiological and pathological processes including neural development, neural regeneration and degeneration, neural plasticity, neuronal apoptosis and neuroinflammation (Antoniou and Borsello, 2012). The activation of JNK signaling pathways is considered to be a blockage of therapeutic success for their effects of promoting inflammation and apoptosis at the early stage of stroke. Therefore, the suppression of these JNK signaling pathways in the early phase has been proved to be neuroprotective (Borsello et al., 2003). However, the delayed inhibition of these pathways, 7 days after stroke, aggravates the neurological outcomes with larger infarction volumes (Murata et al., 2012), indicating that the JNK signaling pathway plays distinguishing roles in different phases of stoke. In a later stage of stroke, neural regeneration and formation of new neural circuits become the primary therapeutic goals. These recovery and reconstructive processes are similar to the neural development when JNK signaling pathways have leading roles in axonal regeneration in both brain development and neural injury repair (Waetzig et al., 2006). What is more, JNK1-knockout mice presented distinctly larger infarct volume, with a higher expression of JNK3 in the penumbra after the permanent cerebral ischemic injury, than that of the wild type, suggesting the potential role of JNK1 in the long-term neural remodeling. A previous study has documented that Netrin-1 binding to DCC can increase endogenous JNK1 activity in primary neurons and that in developing spinal cord Netrin-1 dramatically increases the level of endogenous p-JNK in commissural axon growth cones while JNK1 RNAi can suppress Netrin-1-induced axon attraction and neurite outgrowth (Qu et al., 2013). c-Jun, one of the downstream molecules of JNKs, is essential for axonal regeneration. The above findings indicate that JNK1 is specifically required for the Netrin-1-induced neurite outgrowth. In our study, we also found that AAV-NT-1-mediated Netrin-1 overexpression significantly increased the level of p-JNK1 and p-c-Jun in the peri-infract area after the MCAO. So, we speculate that the activation of the JNK1 pathway may be the potential underlying mechanism for the Netrin-1-induced protective effects after stroke.

Behavioral recovery is a comprehensive embodiment of neural functional recovery after the cerebral ischemia, indicating the degree of neural structural recovery. In the present study, the mNss scores in each group showed no difference at Day 1 and 3, but at Day 7, 10 and 14, rats in the AAV-NT-1 group showed fewer neurological deficits than the AAV-empty group. Of note, these beneficial effects of Netrin-1 were remarkably decreased by inhibiting the JNK1 activity. In accordance with previous studies (Lu et al., 2011, 2012; Sun et al., 2011), the results of the current study suggest that Netrin-1 can facilitate neural functional recovery and reduce neural impairment after stroke. Furthermore, we prove that these effects of Netrin-1 may partly depend on the activity of JNK1/c-Jun signaling pathway.

As a transmembrane protein, DCC binds with its ligand Netrin-1 and participates in the synaptogenesis and axon guidance during CNS development. DCC can also regulate synaptic pasticity in mature mammalian brains by activating Src, enhancing NMDAR function, and is required for NMDAR-dependent long-term potentiation (LTP) (Horn et al., 2013). Furthermore, DCC is expressed in both neurons and astrocytic feet and affects the promoting effects of Netrin-1 on axonal growth after a transient cerebral ischemia (Tsuchiya et al., 2007). In the current study, we found that Netrin-1 overexpression up-regulated the expression of DCC, but had no effect on the relative level of p-DCC, indicating that the activation of JNK signaling pathway may play a role in Netrin-1-mediated axonal regeneration and synaptic plasticity, which may be DCC-dependent, but the specific mechanisms need further exploration. A previous study reported that DCC and p-JNK were co-expressed in the mouse spinal cord, precrossing commissural axons, motor column, and postcrossing commissural axons (Qu et al., 2013). Our immunofluorescence result of co-localization further proves that Netrin-1 binds to DCC to regulate the activities of JNK1 pathway.

Cerebral ischemia can directly cause the destruction of synaptic structure and cut off the neural transmission. Surprisingly, it has been estimated that 1 hour of cerebral ischemia can result in damages of 830 billion synapses and 714 km of myelin (Saver, 2006). Besides, structural and morphological reestablishment is the basis for functional plasticity of cortical circuits (Wilbrecht et al., 2010). The postsynaptic density (PSD) is an electron-dense structure positioned at the postsynaptic side of neurons and is composed of cytoskeletal and scaffold proteins, receptors, adhesion molecules, signaling molecules (Trinidad et al., 2005; Lowenthal et al., 2015). The interactions of PSD components are involved in synaptogenesis, synaptic transmission and synaptic plasticity (Won et al., 2017). After a short period of cerebral ischemia, the size of PSD increases transiently by recruiting several PSD proteins (Hu et al., 1998), leading to an increase of synaptic transmission at the early stage of stroke (Murphy and Corbett, 2009). After that, persistent hypoxic ischemia injury impairs the PSD structure and function (Xu et al., 2009). Ultrastructural parameters, including PSD thickness, length, area, and width of the synaptic cleft, are reliable for quantitative analysis of synaptic plasticity (Jing et al., 2004; Fukunaga et al., 2015). In our study, we assessed the axonal and synaptic ultramicrostructures by TEM at post-ischemia Day 14. In the AAV-NT-1 group, both axonal and synaptic ultrastructures were clear and relatively intact, while these ultrastructures in the AAV-empty and AAV-NT-1 +SP600125 group were fuzzy and disorganized. Furthermore, the results of quantitative analysis of synapses indicate that Netrin-1 promotes PSD reconstruction after stroke via mediating PSD thickness, PSD area and width of the synaptic cleft. Additionally, the suppression of JNK1 signaling pathway can partly reduce these beneficial effects.

Synaptophysin, a biomarker expressed abundantly in the pre-synaptic area, is often measured to quantify synaptic plasticity and synaptogenesis in both neural development and remodeling (Li et al., 2010). Netrin-1 overexpression can up-regulate the expression of SYN and GAP-43 in the spinal cord injury in rats and improve motor and sensory functions (Han et al., 2017). PSD-95 is expressed exclusively in the post-synaptic region of neurons (Hunt et al., 1996) and plays a key role in synaptic plasticity and the stabilization of synaptic changes during the LTP (Meyer et al., 2014), influencing learning and memory. Netrin-1 can significantly enrich PSD-95 and AMPA receptors in cultured cortical neurons and facilitate excitatory synaptogenesis (Goldman et al., 2013; Goldman, 2014).

In our study, we found that after stroke, Netrin-1 overexpression increased the expression level of both SYN and PSD-95, indicating that Netrin-1 can promote synaptic formation in both the pre-synaptic and post-synaptic region. Additionally, the effects of Netrin-1 on synapses were partly abolished by SP600125, suggesting that the JNK1/c-Jun pathway mediates Netrin-1-induced synaptic formation after stroke.

The neurofilaments, known as structural substrates of the axonal cytoskeleton, are considered to be requisite in axonal maintenance and regeneration (Stoll and Muller, 1999). GAP-43 is essential for neurite formation, axon pathway finding, outgrowth of new axons and sustaining of synaptic stability in both neural development and nerve injury (Raff et al., 2002; Stokin et al., 2005; Shen and Meiri, 2013; Mascaro et al., 2013). Studies have showed that GAP-43 contributes to the persistent post-stroke axon sprouting by awakening the neurons in the peri-infarct cortex (Carmichael et al., 2005). Furthermore, in callosal neuritis, GAP-43 is essential for Netrin-1 to stimulate both neurite outgrowth and guidance, while in ventrolateral efferents, it is required only for Netrin-1 to stimulate outgrowth but not guidance (Shen and Meiri, 2013). Therefore, GAP-43 can represent a marker for axonal regeneration. Microtubule-associated protein 2 (MAP-2) is a cytoskeletal protein abundant in dendrites that are closely related to dendritic outgrowth, brunching, remodeling, and synaptic plasticity (Johnson and Jope, 1992). It is often chosen to measure neuronal damage and dendritic restructuring after the cerebral ischemia (Li et al., 1998; Liu et al., 2005). In our study, we adopted NF-200, GAP-43, and MAP-2 to evaluate the capacity of axonal regeneration. We found that Netrin-1 over-expression after stroke promoted axonal outgrowth and regeneration by up-regulating the expression level of GAP-43, NF-200, and MAP-2 and that the suppression of the JNK1/c-Jun pathway reduced the expression of GAP-43 and NF-200 but did not affect the protein level of MAP-2. We speculate that the activation of the JNK1/c-Jun pathway via Netrin-1 over-expression can promote axonal regeneration while there is little effect on dendritic regeneration.

## Conclusion

Taken together, our results indicate that Netrin-1 can promote neural functional recovery after the cerebral ischemia. Its binding with DCC facilitates synaptic formation and axonal regeneration by up-regulating SYN, PSD-95, GAP-43, and MAP-2, and inducing neural ultramicrostructural reconstruction. These effects on neural circuit reestablishment partly depend on the JNK1/c-Jun pathway after the acute stage of stroke. This study extends our understanding of Netrin-1’s role in neural regeneration and provides further evidence for its potential value in the cerebral ischemia therapy.

## Author Contributions

MZ drafted the main manuscript and performed the main experiments. NL and RC conceived and designed the experiments. YZ, XJ, and HC revised the manuscript. JC and PL helped with the establishment of the MCAO model. YL and QY participated in western blotting and immunofluorescence staining experiments. XP and QL were responsible for data analysis. All authors read and approved the final manuscript.

## Conflict of Interest Statement

The authors declare that the research was conducted in the absence of any commercial or financial relationships that could be construed as a potential conflict of interest.

## Supplementary Material

FIGURE S1 Western-blot analysis of the supplementary groups. **(A)** The representative image of western-blot analysis for DCC/p-DCC, p-JNK1/JNK1, p-c-Jun/c-Jun. Rats in each group were sacrificed at Day 14 after the MCAO. **(B)** Western-blot analysis of DCC (*n* = 3); **(C)** Western-blot analysis of p-DCC/DCC (*n* = 3). **(D)** Western-blot analysis of p-JNK1 (*n* = 3), ^∗^*p* < 0.001, as compared with the AAV-empty group, ^#^*p* < 0.001, as compared with the AAV-empty+DMSO group. **(E)** Western-blot analysis of p-c-Jun (*n* = 3), ^∗^*p* < 0.001, as compared with the AAV-empty group, ^#^*p* < 0.001, as compared with the AAV-empty+DMSO group.

FIGURE S2 Western-blot analysis of the supplementary groups. **(A)** The representative images of western-blot analysis for PSD-95 and SYN. Rats in each group were sacrificed at Day 14 after the MCAO. **(B)** Western-blot analysis of PSD-95 (*n* = 3), ^∗^*p* < 0.05, as compared with the AAV-empty group; ^∗^*p* < 0.05, as compared with the AAV-empty+DMSO group. **(C)** Western-blot analysis of SYN (*n* = 3), ^∗^*p* < 0.05, as compared with the AAV-empty group; ^∗^*p* < 0.05, as compared with the AAV-empty+DMSO group.

FIGURE S3 Western-blot analysis of the supplementary groups. **(A)** The representative images of western-blot analysis for NF-200, GAP-43 and MAP-2. Rats in each group were sacrificed at Day 14 after the MCAO. **(B)** Western-blot analysis of NF-200 (*n* = 3). **(C)** Western-blot analysis of GAP-43 (*n* = 3), ^∗^*p* < 0.01, as compared with the AAV-empty group, ^∗^*p* < 0.05, as compared with the AAV-empty+DMSO group. **(D)** Western-blot analysis of MAP-2 (*n* = 3).
