# Supplementary figures and images for: Netrin-1 Promotes Synaptic Formation and Axonal Regeneration via JNK1/c-Jun Pathway after the Middle Cerebral Artery Occlusion
Source: Front Cell Neurosci. 2018 Feb 13;12:13. doi: 10.3389/fncel.2018.00013 (PMC5816818; doi:10.3389/fncel.2018.00013)

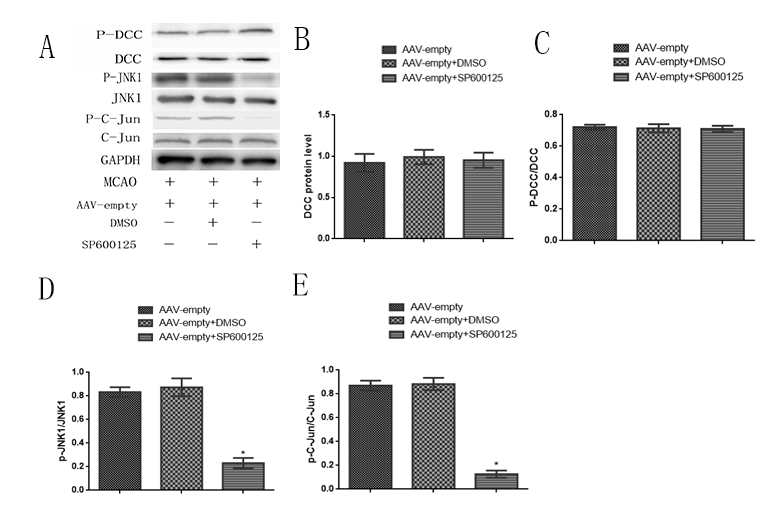

Supplement: FIGURE S1 — Western-blot analysis of the supplementary groups. (A) The representative image of western-blot analysis for DCC/p-DCC, p-JNK1/JNK1, p-c-Jun/c-Jun. Rats in each group were sacrificed at Day 14 after the MCAO. (B) Western-blot analysis of DCC (n = 3); (C) Western-blot analysis of p-DCC/DCC (n = 3). (D) Western-blot analysis of p-JNK1 (n = 3), ∗p < 0.001, as compared with the AAV-empty group, #p < 0.001, as compared with the AAV-empty+DMSO group. (E) Western-blot analysis of p-c-Jun (n = 3), ∗p < 0.001, as compared with the AAV-empty group, #p < 0.001, as compared with the AAV-empty+DMSO group. [file Image_1.TIF]

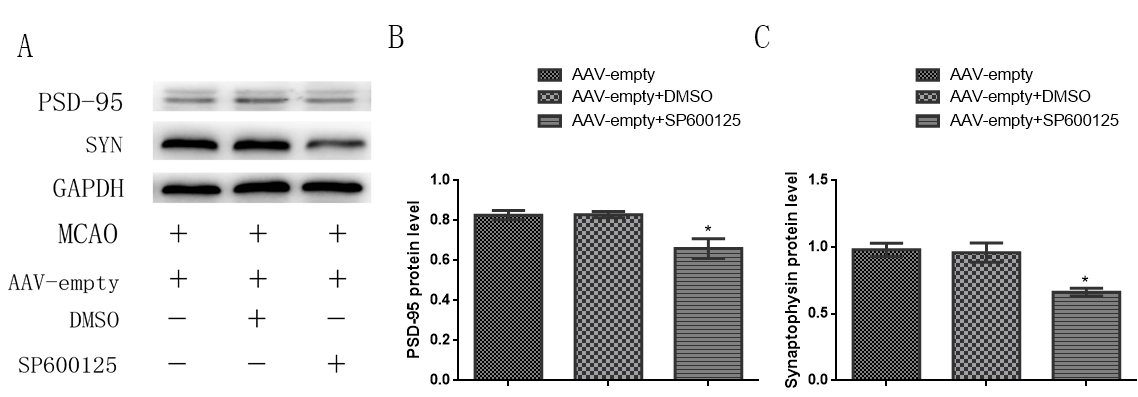

Supplement: FIGURE S2 — Western-blot analysis of the supplementary groups. (A) The representative images of western-blot analysis for PSD-95 and SYN. Rats in each group were sacrificed at Day 14 after the MCAO. (B) Western-blot analysis of PSD-95 (n = 3), ∗p < 0.05, as compared with the AAV-empty group; ∗p < 0.05, as compared with the AAV-empty+DMSO group. (C) Western-blot analysis of SYN (n = 3), ∗p < 0.05, as compared with the AAV-empty group; ∗p < 0.05, as compared with the AAV-empty+DMSO group. [file Image_2.TIF]

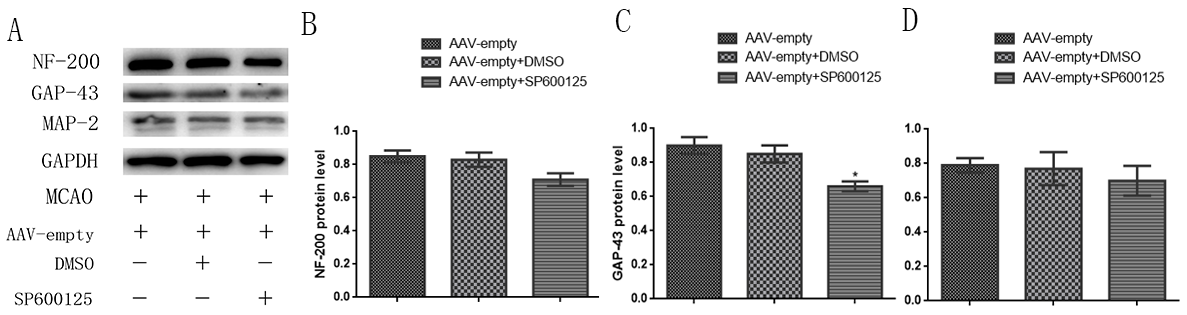

Supplement: FIGURE S3 — Western-blot analysis of the supplementary groups. (A) The representative images of western-blot analysis for NF-200, GAP-43 and MAP-2. Rats in each group were sacrificed at Day 14 after the MCAO. (B) Western-blot analysis of NF-200 (n = 3). (C) Western-blot analysis of GAP-43 (n = 3), ∗p < 0.01, as compared with the AAV-empty group, ∗p < 0.05, as compared with the AAV-empty+DMSO group. (D) Western-blot analysis of MAP-2 (n = 3). [file Image_3.TIF]
